# Measurement of physical activity in urban and rural South African adults: a comparison of two self-report methods

**DOI:** 10.1186/s12889-016-3693-6

**Published:** 2016-09-22

**Authors:** Adewale L. Oyeyemi, Sarah J. Moss, Makama A. Monyeki, Herculina S. Kruger

**Affiliations:** 1 Physical Activity, Sport and Recreation (PhASRec), Faculty of Health Sciences, North-West University, Private Bag X6001, Internal Box 481, Potchefstroom, South Africa; 2 Centre of Excellence in Nutrition, Faculty of Health Sciences, North-West University, Potchefstroom, South Africa

**Keywords:** Baecke physical activity questionnaire, Epidemiology, International physical activity questionnaire, Low-and middle-income countries, Public health guidelines, Surveillance

## Abstract

**Background:**

Due to the large mortality from inactivity-related non-communicable diseases in low- and middle- income countries, accurate assessment of physical activity is important for surveillance, monitoring and understanding of physical (in)activity epidemiology in many of these countries. Research on relative performance of self-report physical activity instruments commonly used for epidemiological research in Africa have rarely been reported. The present study compared estimates of physical activity measured with the International Physical Activity Questionnaire – Short Form (IPAQ-SF) and the Baecke Physical Activity Questionnaire (BPAQ) among urban and rural black South African adults.

**Methods:**

Self-reported physical activity data using the IPAQ-SF and BPAQ were collected from a representative sample of 910 urban and rural black South African adults (age = 59.2 ± 9.5 years, 69.7 % women) participating in the 2015 wave of the Prospective Urban and Rural Epidemiological (PURE) study in the North West Province of South Africa. Between-method relationships (pearson correlations [*r*] and intraclass correlation coefficients [ICCs]) and agreements (Bland-Altman mean difference with 95 % limits of agreement and Kappa coefficient [*k*]) of IPAQ-SF and BPAQ variables were estimated. Sensitivity and specificity of the BPAQ relative to the IPAQ-SF to classify individuals according to the international guidelines for sufficient physical activity were calculated using chi-square statistics.

**Results:**

Correlations between IPAQ-SF scores and BPAQ indices were small (*r* = 0.08–0.18; ICCs = 0.09–0.18) for BPAQ leisure and sport indices, moderate-to-large for work index (*r* = 0.42–0.59; ICCs = 0.40–0.62) and total physical activity index (*r* = 0.52–0.60; ICCs = 0.36–0.51). Between methods mean difference for total physical activity was large (1.85 unit), and agreement in physical activity classifications was poor to moderate (k = 0.16–0.44). The sensitivity of the BPAQ to identify sufficiently active people from the IPAQ-SF was very good (98 %), but its specificity to correctly classify insufficiently active people was weak (23 %).

**Conclusion:**

Notable disparities in physical activity estimates between methods suggest that utilization of IPAQ-SF and BPAQ for surveillance and epidemiology studies in Africa should depend on research questions and population to be studied. Future studies with objective measures are needed to confirm the relative validity between the two instruments.

## Background

Physical inactivity is one of the most important preventable causes of the over 38 million worldwide deaths related to non-communicable diseases (NCDs) [1]. The majority of these deaths (28 million deaths) occurs in low- and middle- income countries (LMICs) where the understanding of evidence based strategies for increasing physical activity is poor [1, 2]. Improving physical activity research in LMICs is a top priority for reducing the pandemic of physical inactivity globally [3, 4]. However, for physical activity research and surveillance to progress in LMICs, it is important to identify appropriate measures that are culturally sensitive and relevant to habitual physical activity behaviors of people in these countries.

Numerous objective and subjective self-report methods are available for measuring habitual physical activity behaviors. Objective measures such as pedometers, accelerometers and heart rate monitors have been advocated for use in physical activity epidemiological studies in high income countries [5–8], because they offer more precise estimates of the volume of physical activity and remove some limitations (e.g. social desirability and recall bias) associated with self-report methods [9–11]. However, self-report measures are very often used in LMICs because the utilization of objective methods is particularly challenging in most of the region countries [12–15]. Of note is the low feasibility of using accelerometers in large epidemiological studies in Africa because of limited financial resources and expertise [15–17]. Self-report represents the most convenient and feasible method for physical activity surveillance in Africa because of their low cost, ease of use and ability to capture domain and context specific behaviors [14–17]. However, to be appropriate for research, self-report measures of physical activity must be locally sensitive, valid and able to generate meaningful and internationally comparable data [16, 18].

The Baecke physical activity questionnaire (BPAQ) and the international physical activity questionnaire (IPAQ) were developed in western high income countries but are commonly used self-report measures of physical activity in South Africa [19–22]. The BPAQ was designed as a brief measure to evaluate habitual physical activity levels and it captures activity done over the past 12 months at work, during sports and at leisure time [23]. This instrument has been modified and extensively validated [24–27], and used in epidemiologic studies in many regions of the world including Africa [20, 21, 28–30]. However, the BPAQ expresses physical activity indices in arbitrary units that are difficult to express in energy expenditure equivalence [23, 31] and may not be able to estimate the prevalence of individuals complying with the public health recommendations of at least 150 min/week of physical activity [32, 33].

More recently, the IPAQ was developed as an instrument for standardizing the assessment of prevalence of physical activity and for assessing population comparable levels of physical activity in different countries and cultures across the world [16]. Similar to the BPAQ, the IPAQ has been validated and extensively adopted for research purposes in many parts of the world and some African countries [17, 22, 34–37]. The IPAQ has two versions (the short and the long forms) that were designed for different purposes. Because of its feasibility for population surveillance and ability to quantify the prevalence of individuals meeting physical activity recommendations [16, 38], the IPAQ-Short Form (SF) is often used for assessment of physical activity in large scale epidemiological studies [13, 39–41]. However, unlike the BPAQ, the IPAQ-SF questions about the time spent at different intensities of activities, is not domain specific, does not provide context specific information on physical activity behaviors and is based on shorter recall (last 7 days vs past 12 months). Moreover, the application of IPAQ may pose challenge to some people in LMICs where study participants have been reported to find it difficult to estimate time spent in different activities [19, 42]. Thus, for physical activity research in LMICs it is important to understand if physical activity estimates from the IPAQ-SF and the BPAQ could be compared or are related and if the two measures could be used interchangeably in research of similar design.

Until now, only one study has attempted investigating such comparison and found modest relationship between total physical activity from the BPAQ and IPAQ-SF among obese adults from European countries [31]. However, questions still remain about relationships and agreements between subcomponents of physical activity assessed by the BPAQ and IPAQ-SF in the general population. For example, how is the moderate-to-high physical activity category on BPAQ able to correctly predict the criteria for meeting moderate- to vigorous intensity physical activity (MVPA) guidelines from IPAQ-SF, and to what extent is the relationship between IPAQ-SF variables and BPAQ indices due to measurement error or actual population phenomenon. This kind of knowledge is important for surveillance and monitoring in physical activity epidemiology, and could improve our understanding of how physical activity instruments could complement each other or be used differently in research of similar designs. Given the large financial implication that may be associated with the use of objective measures, identifying the strength and direction of relationships between all subcomponents of physical activity assessed by IPAQ-SF and BPAQ may shed some light on how constructs and items-wording on both questionnaires could be improved for the purpose of surveillance in LMICs of Africa. Since results of studies aimed to improve physical activity measurement among populations in western high-income countries may not be directly extrapolated to populations or ethnic groups in LMICs [12], it is important to conduct African specific studies.

Studies comparing the performance among physical activity questionnaires have rarely been conducted in Africa [22]. Therefore, the aim of the present study was to compare physical activity estimates from the IPAQ-SF and the BPAQ among urban and rural black South African adults. Based on previous research showing evidence of relationship between components of physical activity from different questionnaires [31, 43, 44], the formulated hypothesis for the present study was that physical activity data obtained by the IPAQ-SF and BPAQ would be related.

## Methods

### Study design

Cross-sectional data were obtained from participants of the third wave of the Prospective Urban and Rural Epidemiological (PURE) study, which was conducted in the North West Province of South Africa in 2015. The methods and population characteristics of the PURE study have been described fully elsewhere [45, 46]. Briefly, the PURE study was an international multi-country prospective epidemiological survey that aimed to follow up the health status and health related lifestyles of adults for 10 years from communities in four low income-, ten middle-income and three high-income countries. Within each of the 17 participating countries, representative samples of adults initially aged 35–70 years who reside in rural and urban communities were selected to participate in the study [45, 46]. South Africa is one of the middle-income countries where the PURE study was conducted from 2005 to 2015.

### Participants and procedure

Participants were 910 urban and rural black South African adults that agreed to participate and provided usable data in the 2015 wave of the North West Province, South African leg of the PURE study. The population for the 2015 wave of PURE study in North West Province of South Africa consisted of 1280 black Setswana speaking South African men (*n* = 437) and women (843) who provided baseline data in 2005, and living in either urban or rural areas of the Province. Participants were approached for the study’s follow-up interest through door-to-door visits of all previously enumerated households, and were invited to a central clinic for data collection in both urban and rural areas. Physical activity questionnaires were administered by trained research staff and all participants were interviewed in their preferred language (Afrikaans, English or Tswana). All participants completed both questionnaires at the same time but the order of administration was based on consecutive alternation during interview contact (i.e., all odd interviewed participants completed the BPAQ first, while all even interviewed participants completed the IPAQ first). Participants were included in the survey if they agreed to participate in the 2015 data collection. A total of 923 participants out of the 1280 at baseline (347 lost to follow-up) were eligible for the 2015 study, and 910 participants (276 men, 634 women) provided complete and usable physical activity surveys, giving a response rate of 98.6 %. Participants who were unwilling to be interviewed (*n* = 5) or had a disability that prevented independent ambulation (*n* = 4) or provided incomplete physical activity data (*n* = 4) were excluded from the 2015 study.

### Measures

#### Baecke physical activity questionnaire

The validated BPAQ for South Africa was used for this study [19]. The questionnaire consists of 21 questions organised into three sections: physical activity at work (Questions 1–11), sport during leisure time (Questions 12–18), and physical activity during leisure excluding sport (Questions 19–21) [23]. A walking index could be created by adding scores of items “At work I walk” and “During leisure time I walk” from the work and leisure (excluding sport) sections, respectively. The questionnaire defined three levels of occupational/work physical activity, namely low (e.g., clerical work, driving, shop keeping, teaching, studying, housework, and occupations with a university education), middle (e.g., factory work, carpentry, farming), and high (e.g., dock work, construction work, manual labour). Similarly, the questionnaire categorised sports into three levels: low (e.g., billiards, sailing, golf), middle (e.g., badminton, cycling, dancing, tennis), and high (e.g., boxing, rugby, football, rowing). Questions in each of the four indices (work, sports, leisure and walking) were scored on a five-point Likert scale, ranging from “1 = never” to “5 = always” or “5 = very often.” The sum of the four indices gives an indicator of total physical activity index (PAI) [23]. The three-months test-retest reliability of the work index (Pearson *r* = 0.88), sport index (Pearson *r* = 0.81) and leisure-time index (Pearson *r* = 0.74) of the original developed BPAQ tested among the Dutch adults is high [23]. Criterion validity for the BPAQ total index as against the energy expenditure from a 3-day diary (Pearson *r* = 0.61) among Chinese adults and from the doubly labelled water (Spearman *r* = 0.54) among Dutch adults is good [24, 25]. The BPAQ is reliable (Spearman *r* = 0.88) and valid when compared with 24-h activity recalls among adults in South Africa [19].

#### International Physical Activity Questionnaire (IPAQ) - short version

In contrast to the BPAQ (Table 1), the short, interviewer-administered IPAQ used in this study contained seven questions that measure the frequency (days/week) and duration (minutes/day) of participation in vigorous-and moderate-intensity activities, and walking in bouts of at least 10 min in the last 7 days [16, 47]. Physical activity outcomes from the IPAQ-SF for this study were weekly minutes time spent in vigorous-intensity physical activity, moderate-intensity physical activity, walking, moderate- and vigorous-intensity physical activity (MVPA), sedentary time and total physical activity. To determine the total physical activity level, scores for each of vigorous, moderate, and walking activity were calculated in MET-minutes per week by multiplying the MET intensity for each activity by minutes per week spent in each activity. One MET represents the energy expended while sitting quietly at rest and is equivalent to 3.5 ml/kg/min of VO_2_ [33]. The MET intensities used to score IPAQ were vigorous (8METs), moderate (4METs) and walking (3.3 METs) [16, 47]. To estimate the number of participants meeting the international public health recommendations of 150 min per week of MVPA [32, 33], a score for MVPA was computed by summing minutes of time per week of moderate- and vigorous-intensity activity. However, given that many participants in this study were old adults (mean age = 59 years), MVPA outcomes were further categorized as (1) inactive: less than 30 min/week of MVPA, (2) insufficiently active: 30–149 min/week of MVPA, and (3) sufficiently active: 150 min/week or more MVPA. In a 12-country (including South Africa) reliability and validity study among adults, the IPAQ-SF demonstrated good evidence of one-week test-retest reliability (spearman *r* = 0.70–0.97), and its criterion validity for total physical activity minutes per week as measured against accelerometer total counts was acceptable (Spearman *r* = 0.23) [16].

**Table 1:** Information from the Baecke Physical Activity Questionnaire (BPAQ) and the International Physical Activity Questionnaire-Short Form (IPAQ-SF)

Baecke physical activity questionnaire	International physical activity questionnaire
Work index	Vigorous physical activity
1. What is your main occupation? *low level/middle level/high level*	1. During the last 7 days, on how many days did you do vigorous physical activities like heavy lifting, digging, aerobics, or fast bicycling? *___ Days per week*
2. At work I sit? *never/seldom/sometimes/often/always*	2. How much time did you usually spend doing vigorous physical activities on one of those days? *_ _ Hours per day; _ _ Minutes per day*
3. At work I stand? *never/seldom/sometimes/often/always*	Moderate physical activity
4. At work I walk? *never/seldom/sometimes/often/always*	3. During the last 7 days, on how many days did you do moderate physical activities like carrying light loads, bicycling at a regular pace or double tennis? *___ Days per week*
5. At work I lift heavy loads? *never/seldom/sometimes/often/always*	3. How much time did you usually spend doing moderate physical activities on one of those days? *_ _ Hours per day; _ _ Minutes per day*
4. At work I walk? *never/seldom/sometimes/often/always*	Walking for recreation, exercise, sport, travel, at home or work
5. At work I am tired? *never/seldom/sometimes/often/always*	5. During the last 7 days, on how many days did you do moderate physical activities? *___ Days per week*
6. At work I sweat? *never/seldom/sometimes/often/always*	6. How much time did you usually spend doing moderate physical activities on one of those days? *_ _ Hours per day; _ _ Minutes per day*
7. If you work away from home, how do you get to work? *walk/cycle/car/taxi*	Sedentary time
8. How long does it take you to walk/cycle to work? *0*–*15 min/16–30 min/31–60 min/1–2 h*	7. During the last 7 days, on how much time did you usually spend sitting (at work, at home, during leisure time reading or watching TV) on a week days did you do moderate physical activities? *_ _Hours per week; _ _Minutes per week*
9. What is your usual pace if you walk or cycle to work? *casual strolling/fairly brisk/brisk or fas*t	
Sport index	
10. Do you play sport? *yes/no*	
11. Which sport do you play most frequently? *low level/middle level/high level*	
12. How many hours per week do you practice the sport? *<1 h/ 1–2 h/ 2–3 h/ 3–4 h/ >4 h*	
13. How many months a year do you practice the sport? *<1/ 1–3/ 4–6/ 7–9/ >9 months*	
14. If you play a second sport, which is it? *low level/middle level/high level*	
15. How many hours per week do you practice the second sport? *<1 h/ 1–2 h/ 2–3 h/ 3–4 h/ >4 h*	
16. How many months a year do you practice the second sport? *<1 / 1–3/ 4–6/ 7–9/ >9 months*	
Leisure index	
17. During leisure time I watch TV/ do sitting activities (read, needle-work, play cards) *never/seldom/sometimes/often/always*	
18. During leisure time I walk/ do standing activities (gardening/housework) *never/seldom/sometimes/often/always*	
19. Other leisure activities……………… *never/seldom/sometimes/often/always*	

#### Demographics and anthropometric measurement

Demographic data included participants’ age and gender. Participants’ height and weight were measured with calibrated instruments (Precision Health Scale, A & D Company, Japan; Seca Stadiometer, Hamburg, Germany) and body mass index (BMI) was calculated as weight divided by height squared (kg/m^2^).

### Statistical analyses

Physical activity data from IPAQ-SF were cleaned and edited using the recommendations in the IPAQ data processing guidelines [47]. To limit unrealistic high values, all walking, moderate, and vigorous physical activity variables exceeding 3 h/day were truncated to be equal to 3 h (for 56 participants), and when time variables were lower than 10 min/day, they were recoded to zero (for 83 participants). Descriptive statistics of mean, standard deviation and frequencies were calculated for the characteristics of the participants and the physical activity estimates from IPAQ-SF and BPAQ. Because physical activity levels are generally different between men and women and by ‘urbanicity’ [48], mean group differences for continuous variables by gender (men vs women) and study location (urban area vs rural area) were examined by independent *t*-test, and for dichotomous variables by chi-square statistics. Relationships between physical activity estimates from the IPAQ-SF and BPAQ were determined using Pearson correlation coefficients. Intraclass correlation coefficients (ICCs) were also calculated. The ICC models used were two-way mixed effects models appropriate for the assessment of validity [49], where allowance is made for difference in data collection. The ICCs calculations were based on single measurements and consistency agreements rather than absolute agreements definitions because the physical activity outcomes from the various assessment instruments did not share a common metric (e.g., minutes/week or MET-minutes/week) and could only be simply correlated rather than be directly compared [49–51]. Strength of relationships was determined using Cohen’s effect sizes for correlation coefficients (*r* = 0.10 to 0.30, small; *r* = 0.31 to 0.50, moderate; *r* > 0.50, large) [52]. The Bland and Altman plot with mean difference and 95 % limits of agreement was used to determine the magnitude of error (disagreement) in total physical activity estimates between methods [53]. Cross-tabulation with chi-square statistics was used to explore the sensitivity and specificity of the BPAQ physical activity categories to determine the proportion of individuals meeting or not meeting the current public health physical activity recommendations according to the IPAQ-SF classifications [32, 33]. Cohen’s Kappa statistics (*k*) was computed as a measure of agreement between physical activity classifications from the two questionnaires. Because physical activity estimates from the IPAQ-SF and BPAQ were skewed, log-transformation of the original variables was used to improve their normality in the correlation and Bland-Altman analyses. Raw data were used to calculate the descriptive statistics for the physical activity variables (both IPAQ and BPAQ) shown in the Result section and Tables. All statistical analyses were performed using Statistical Package for the Social Science (SPSS), version 18.0 for windows (SPSS Inc., Chicago, Illinois, USA) and the level of significance was set at *P* ≤ 0.05.

## Results

The characteristics of the participants are shown in Table 2. Overall, the participants comprised of 69.7 % women and 30.3 % men, with mean age of 59.2 (Standard Deviation [SD] = 9.5) years and body mass index of 26.2 (SD = 7.5) kg/m^2^. The majority of the participants were from the rural areas (58.2 %). The urban areas participants were older than those from the rural areas (*P* = 0.002). Women from both the rural and urban areas had higher BMI compared to the men from both areas (*P* < 0.001).

**Table 2:** Characteristics of the participants

Variable	*N*	All	Men (*n* = 276, 30.3 %)	Women (*n* = 634, 69.7 %)	*P*-value
		Mean (SD)	Mean (SD)	Mean (SD)	(Gender)
Age (years)	910	59.2 (9.5)	59.7 (9.6)	59.1 (9.4)	0.398
Rural	530	58.5 (8.9)*	59.4 (9.7)	58.0 (8.5)	0.095
Urban	380	60.4 (10.2)	59.9 (9.6)	60.6 (10.4)	0.574
BMI (kg/m^2^)	900	26.2 (7.5)	21.8 (4.9)	28.1 (7.7)	<0.001
Rural	526	25.9 (7.2)	21.9 (4.9)	27.6 (7.4)	<0.001
Urban	374	26.6 (7.9)	21.8 (4.9)	28.9 (8.1)	<0.001

*Significant difference between rural and urban sample (*P* = 0.002)

Physical activity variables from the BPAQ and IPAQ-SF are presented in Table 3. Only the sport index (higher in urban than rural participants) was significantly different by location (*P* = 0.014) on the BPAQ. Walking (*P* < 0.001), sport (*P* = 0.021), leisure (*P* < 0.001) and total physical activity indices on BPAQ were higher in men than women. The BPAQ significantly classified (*P* < 0.001) more men (26.4 %) than women (8.5 %) to be highly active but more women (82.8 %) than men (58.4 %) to be moderately active. For the IPAQ-SF, urban participants were significantly (*P* < 0.001 for all) more active than the rural participants during all intensities of physical activity (except for vigorous-intensity physical activity). Men spent more time in vigorous-intensity (*P* < 0.001), moderate-intensity (*P* = <0.001), walking (*P* = 0.005), MVPA (*P* < 0.001) and total physical activity (*P* = 0.001) than women. More men than women (63.8 % vs 57.3 %, *P* = 0.055), and more urban (*n* = 257, 67.6 % vs *n* = 282, 53.2 %, *p* < 0.001) than rural participants were classified by IPAQ-SF as sufficiently active to meet the international public health guidelines.

**Table 3:** Physical activity estimates from the Baecke questionnaire and IPAQ-SF

Variable	All	*P*-value	Men	Women	*P*-value
	Mean (SD)	(Location)	Mean (SD)	Mean (SD)	(Gender)
Beacke questionnaire					
Work index	2.8 (0.9)		2.7 (1.1)	2.8 (0.7)	0.103
Rural	2.7 (0.9)	0.107	2.6 (1.1)	2.8 (0.8)	0.113
Urban	2.8 (0.8)		2.8 (1.1)	2.9 (0.6)	0.506
Walking index	0.3 (0.8)		0.5 (1.0)	0.2 (0.6)	<0.001*
Rural	0.3 (0.8)	0.246	0.6 (1.1)	0.2 (0.7)	<0.001*
Urban	0.3 (0.7)		0.4 (0.8)	0.2 (0.6)	0.070
Sport index	0.1 (0.4)		0.1 (0.5)	0.0 (0.3)	0.021*
Rural	0.0 (0.3)	0.014*	0.0 (0.2)	0.0 (0.3)	0.503
Urban	0.1 (0.5)		0.2 (0.8)	0.0 (0.3)	0.002*
Leisure index	1.9 (0.9)		2.1 (0.9)	1.8 (0.8)	<0.001*
Rural	1.9 (0.9)	0.909	2.1 (1.0)	1.8 (0.8)	<0.001*
Urban	1.9 (0.9)		2.1 (0.9)	1.8 (0.9)	<0.001*
Total PA index	5.0 (1.7)		5.5 (2.4)	4.9 (1.3)	<0.001*
Rural	5.0 (1.8)	0.423	5.5 (2.4)	4.8 (1.4)	<0.001*
Urban	5.1 (1.6)		5.5 (2.1)	4.9 (1.2)	<0.001*
PA category (%)					
Low PA (%)	97 (10.7)		42 (15.2)	55 (8.7)	<0.001*
Moderate PA (%)	686 (75.3)		161 (58.4)	525 (82.8)	
High PA (%)	127 (14.0)		73 (26.4)	54 (8.5)	
IPAQ-SF					
Vigorous PA (min/wk)	50.9 (130.4)		84.3 (174.6)	36.4 (102.3)	<0.001*
Rural	56.6 (136.6)	0.118	95.1 (167.4)	40.5 (117.8)	<0.001*
Urban	42.9 (121.0)		70.1 (183.5)	30.6 (74.7)	0.003*
Moderate PA (min/wk)	272.3 (288.4)		336.4 (335.7)	244.4 (260.6)	<0.001*
Rural	229.9 (264.3)	<0.001*	302.2 (324.9)	199.6 (227.9)	<0.001*
Urban	331.4 (309.8)		381.5 (345.7)	308.6 (289.8)	0.033*
Walking (min/wk)	240.9 (255.7)		277.1 (272.9)	225.2 (246.5)	0.005*
Rural	215.7 (207.5)	<0.001*	249.5 (244.2)	201.4 (188.4)	0.015*
Urban	276.2 (307.0)		313.6 (303.8)	259.1 (308.5)	0.110
Total PA (MET-min/wk)	2291.3 (2188.6)		2934.3 (2744.3)	2011.5 (1829.8)	<0.001*
Rural	2084.2 (2194.4)	0.001*	2792.6 (2761.6)	1786.1 (1829.9)	<0.001*
Urban	2580.3 (2150.3)		3121.2 (2721.7)	2333.6 (1784.2)	0.001*
MVPA (min/wk)	323.1 (358.6)		420.5 (437.5)	280.8 (309.2)	<0.001*
Rural	286.5 (354.7)	<0.001*	397.3 (437.8)	239.9 (301.9)	<0.001*
Urban	374.2 (358.3)		451.1 (437.1)	339.1 (310.5)	0.005*
Sedentary time (min/wk)	297.8 (162.1)		311.4 (190.6)	291.8 (147.8)	0.093
Rural	285.2 (163.9)	0.006*	304.1 (198.1)	277.3 (146.8)	0.085
Urban	315.2 (158.2)		321.1 (180.6)	312.5 (147.1)	0.625
Activity group (%)					
Inactive	150 (16.5)		57 (20.7)	93 (14.7)	<0.001*
Not sufficiently active (%)	221 (24.3)		43 (15.6)	178 (28.1)	
Sufficiently active (%)	539 (59.2)		176 (63.8)	363 (57.3)	

*SD* standard deviation, *PA* physical activity, *MVPA* moderate-to-vigorous physical activity, *min/wk* minutes per week

*significant *P*-value

Table 4 shows the correlations between IPAQ-SF variables (MVPA, walking, total physical activity and sitting) and BPAQ indices (work, walking, sports, leisure and total physical activity). Significant relationships (*P* < 0.01) were observed for several outcomes between methods. The correlations between all IPAQ-SF variables (except walking) with BPAQ indices were small for leisure and sport indices (*r* = 0.08–0.18; ICCs = 0.09–0.18) and moderate- to- large for work index (*r* = 0.42–0.59; ICCs = 0.40–0.62) and total physical activity index (*r* = 0.52–0.60; ICCs = 0.36–0.51). Similar small to large correlations were observed by gender, but correlation values for all relationships between the two questionnaires tended to be higher for the male participants (Table 4). For location, correlation coefficients values between outcomes from both methods were higher in rural participants, except for the relationships between IPAQ-SF total physical activity and BPAQ sport index, IPAQ-SF walking and BPAQ walking index and IPAQ-SF sedentary time and BPAQ leisure index, for which correlation coefficients values were higher in urban participants (Table 5).

**Table 4:** Correlations between physical activity estimates from the Baecke Questionnaire and IPAQ-SF for Overall Sample and by Gender

Variables	All (*N* = 910)			Men (*n* = 276)			Women (*n* = 643)		
	*R*	ICC	95 % CI	*r*	ICC	95 % CI	*r*	ICC	95 % CI
Baecke vs IPAQ total PA									
Work index	0.54**	0.62	0.57, 0.66	0.63**	0.73	0.66, 0.79	0.49**	0.53	0.46, 0.60
Walking index	0.25**	0.39	0.31, 0.47	0.29**	0.44	0.29, 0.56	0.22**	0.34	0.23, 0.43
Leisure index	0.14**	0.15	0.03, 0.25	0.21**	0.21	−0.01, 0.37	0.10*	0.10	−0.06, 0.23
Sport index	0.11**	0.07	0.01, 0.13	0.14**	0.10	−0.17, 0.22	0.07	0.04	−0.04, 0.12
Total PA index	0.55**	0.43	0.35, 0.50	0.62**	0.53	0.41, 0.63	0.50**	0.35	0.24, 0.41
Baecke vs IPAQ MVPA									
Work index	0.59**	0.46	0.37, 0.51	0.71**	0.57	0.45, 0.66	0.50**	0.35	0.24, 0.44
Walking index	0.20**	0.23	0.12, 0.32	0.29**	0.32	−0.13, 0.46	0.15**	0.17	0.03, 0.29
Leisure index	0.13**	0.10	−0.05, 0.19	0.30**	0.15	−0.07, 0.33	0.05	0.03	−0.13, 0.17
Sport index	0.11**	0.10	−0.06, 0.19	0.16**	0.15	−0.12, 0.30	0.08	0.05	−0.12, 0.18
Total PA index	0.60**	0.36	0.26, 0.45	0.73**	0.46	0.29, 0.59	0.49**	0.29	0.15, 0.40
Baecke vs IPAQ walking									
Work index	0.38**	0.46	0.38, 0.52	0.43**	0.55	0.42, 0.64	0.36**	0.40	0.30, 0.49
Walking index	0.19**	0.30	0.20, 0.38	0.22**	0.34	0.17, 0.48	0.16**	0.25	0.13, 0.36
Leisure index	0.10*	0.10	−0.05, 0.19	0.11	0.11	−0.14, 0.29	0.06	0.06	−0.10, 0.19
Sport index	0.08*	0.09	−0.03, 0.20	0.10	0.14	−0.09, 0.32	0.05	0.05	−0.10, 0.18
Total PA index	0.38**	0.30	0.20, 0.38	0.42**	0.37	0.21, 0.51	0.37**	0.24	0.12, 0.35
Baecke vs IPAQ sedentary									
Work index	−0.42**	−0.40	−0.45, −0.34	−0.52**	−0.63	−0.69, −0.55	−0.33**	−0.38	−0.44, −0.31
Walking index	−0.28**	−0.22	-.028, −0.16	−0.43**	−0.33	−0.43, −0.22	−0.17**	−0.14	−0.22, −0.06
Leisure index	−0.18**	−0.18	−0.24, −0.12	−0.25**	−0.27	−0.38, −0.16	−0.13**	−0.12	−0.20, −0.05
Sport index	−0.18**	−0.18	−0.25, −0.12	−0.28**	−0.25	−0.35, −0.16	−0.13**	−0.13	−0.20, −006
Total PA index	−0.52**	−0.51	−0.55, −0.46	−0.64**	−0.63	−0.69, −0.55	−0.42**	−0.58	−0.44, −0.31

*r* Pearson correlation coefficients, *MVPA* moderate-to-vigorous physical activity, *PA* physical activity, *CI* confidence interval

*P- value significant at <0.05

**P value significant at <0.01

**Table 5:** Correlations between physical activity estimates from the Baecke questionnaire and IPAQ-SF for rural and urban participants

Variable	Rural participants (*n* = 530)			Urban participants (380)		
	*R*	ICC	95 % CI	*R*	ICC	95 % CI
Baecke vs IPAQ total PA						
Work index	0.59**	0.67	0.61, 0.72	0.40**	0.49	0.38, 0.59
Walking index	0.27**	0.40	0.29, 0.49	0.24**	0.38	0.25, 0.49
Leisure index	0.15**	0.16	−0.01, 0.28	0.12*	0.15	−0.04, 0.31
Sport index	0.07	0.03	−0.05, 0.12	0.14**	0.11	0.01, 0.21
Total PA index	0.61**	0.45	0.35, 0.54	0.44**	0.39	0.26, 0.50
Baecke vs IPAQ MVPA						
Work index	0.61**	0.48	0.39, 0.56	0.57**	0.40	0.26, 0.51
Walking index	0.21**	0.24	0.10, 0.36	0.19**	0.22	0.05, 0.37
Leisure index	0.14**	0.08	−0.09, 0.23	0.13**	0.08	−0.13, 0.25
Sport index	0.08	0.04	−0.14, 0.19	0.13**	0.11	−0.09, 0.27
Total PA index	0.61**	0.37	0.24, 0.49	0.57**	0.36	0.19, 0.48
Baecke vs IPAQ walking						
Work index	0.45**	0.51	0.42, 0.59	0.21**	0.29	0.13, 0.42
Walking index	0.18**	0.28	0.14, 0.39	0.21**	0.34	0.19, 0.46
Leisure index	0.05	0.08	−0.09, 0.22	0.11*	0.10	−0.11, 0.26
Sport index	0.08	0.04	−0.14, 0.19	0.08	0.16	−0.03, 0.31
Total PA index	0.44**	0.32	0.19, 0.42	0.28	0.25	0.09, 0.39
Baecke vs IPAQ sedentary						
Work index	−0.44**	−0.53	−0.59, −0.46	−0.42**	−0.47	−0.55, −0.39
Walking index	−0.30**	−0.24	−0.32, −0.16	−0.25**	−0.20	−0.29, −0.10
Leisure index	−0.15**	−0.15	−0.24, −0.05	−0.25**	−0.20	−0.28, −0.12
Sport index	−0.21**	−0.24	−0.34, −0.15	−0.15**	−0.14	−0.23, −0.06
Total PA index	−0.56**	−0.53	−0.59, −0.46	−0.50**	−0.47	−0.55, −0.39

*r* Pearson correlation coefficients, *MVPA* moderate-to-vigorous physical activity, *PA* physical activity, *CI* confidence interval

*P- value significant at <0.05

**P value significant at <0.01

Table 6 shows the sensitivity and specificity results. The sensitivity of the BPAQ (ability of the BPAQ moderate-to high physical activity category) to correctly identify sufficiently active individuals according to the MVPA classifications from the IPAQ-SF was 98 % (i.e., 528 out of 539 were accurately identified from BPAQ as meeting the IPAQ guidelines). Whereas, while the specificity (ability of the BPAQ low physical activity category) to correctly identify those not meeting the current public health guidelines (150 min/week of MVPA) according to the IPAQ-SF was 23 % (or 86 out of 371) (*x*^2^ = 103.12, *P* < 0.001), it was 48.7 % (or 73 out of 150) (*x*^2^ = 319.47, *P* < 0.001) when the IPAQ-SF guideline of less than 30 min/week of MVPA (inactivity) was applied (Not shown in Table). Thus, the specificity of BPAQ low physical activity category to correctly identify the IPAQ insufficiently active (less than 150 min/week MVPA) and inactive (less than 30 min/week MVPA) categories was weak. Using the Landis and Koch guidelines [54], between methods agreement in physical activity classifications from the two questionnaires was fair (*k* =0.24) for all participants combined. Kappas showed poor to moderate agreement (*k* = 0.16–0.44) for group stratified by sex and rural/urban areas (Table 6).

**Table 6:** Number of individuals from Baecke questionnaire meeting physical activity guidelines by IPAQ-SF and agreement for their respective categories

	PA guidelines by IPAQ-SF				Agreement	
PA Categories by Baecke	Meet guidelines	Not meet guidelines	Sensitivity	Specificity	%	Kappa
	*N* (%)	*N* (%)				
Total						
Moderate-to-high PA	528 (64.9)	285 (35.1)	0.98	0.23	68	0.24
Low PA	11 (11.3)	86 (88.7)				
Men						
Moderate-to-high PA	174 (74.4)	60 (25.6)	0.99	0.40	78	0.44
Low PA	2 (4.8)	40 (95.2)				
Women						
Moderate-to-high PA	354 (61.1)	225 (38.9)	0.97	0.17	63	0.16
Low PA	9 (16.4)	46 (83.6)				
Rural						
Moderate-to-high PA	278 (72.5)	190 (27.5)	0.99	0.23	63	0.25
Low PA	4 (6.5)	58 (93.5)				
Urban						
Moderate-to-high PA	250 (72.5)	95 (27.5)	0.97	0.23	73	0.25
Low PA	7 (20.0)	28 (80.0)				

*PA* physical activity

**Fig. 1:**
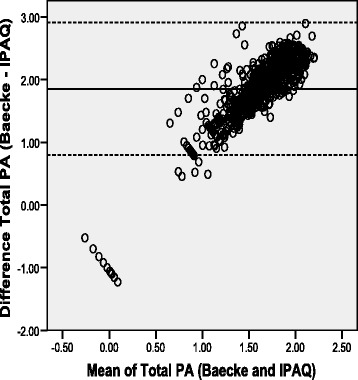
Bland-Altman plot for total physical activity from the Baecke and IPAQ-SF for overall Sample (Mean Difference: 1.85 +/− 2SD = 0.79 to 2.91)

**Fig. 2:**
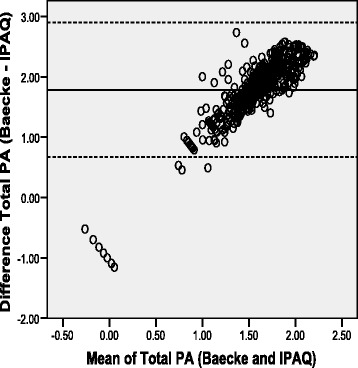
Bland-Altman plot for total physical activity from the Baecke and IPAQ-SF for rural Sample (Mean Difference: 1.78 +/− 2SD = 0.67 to 2.90)

**Fig. 3:**
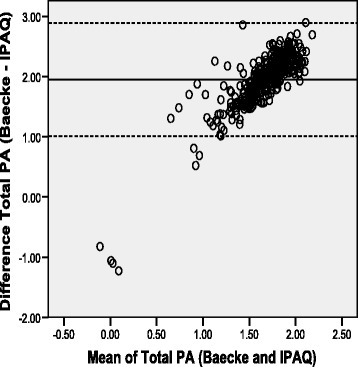
Bland-Altman plot for total physical activity from the Baecke and IPAQ-SF for urban sample (Mean Difference: 1.95 +/− 2SD = 1.01 to 2.89)

Figures 1, 2 and 3 shows the Bland-Altman plots for the agreement between IPAQ-SF and BPAQ total physical activity scores. For the overall sample, the mean difference between methods was 1.85 unit (mean difference significantly higher than zero [*P* < 0.001]) but the variation in 95 % limits of agreement (0.79 to 2.91) was reasonable. Similar large mean differences but acceptable 95 % limits of agreements were recorded for both the rural (1.78 unit, 0.67 to 2.90) and urban (1.95 unit, 1.01 to 2.89) participants. However, notable proportional bias could be observed from the shapes of the three graphs indicating that the differences (i.e., error) between the IPAQ-SF and BPAQ scores increased as the mean scores of the two methods increased (*R*^2^ = 0.81, for overall sample).

## Discussion

This study compared self-reported physical activity estimates from the IPAQ-SF and the BPAQ among urban and rural black South African adults. The findings revealed modest correlations between variables from the IPAQ-SF and BPAQ, and highlighted notable disparities in physical activity estimates between methods. The sensitivity of the BPAQ to correctly identify sufficiently active individuals meeting the international public health guidelines from the IPAQ-SF was good but its specificity to correctly identify insufficiently active individuals was weak.

Few studies could be found that directly compared estimates between physical activity questionnaires [31, 43, 44]. In a study comparing physical activity prevalence estimates among US adults, the Behavioral Risk Factor Surveillance System (BRFSS) physical activity questionnaire and the IPAQ-SF were found to demonstrate fair to moderate correlation [43]. Similar to our finding, Tehard et al. [31] reported modest correlation between total physical activity from the IPAQ-SF and BPAQ among European obese participants. However, our findings seem to advance that other physical activity outcomes from the IPAQ-SF (e.g., MVPA, walking and sitting) were related in the expected direction with all the derived physical activity indices from the BPAQ. Of interest was that the IPAQ sitting item (an aggregate measure of sedentariness) was inversely related with all physical activity indices from the BPAQ, suggesting that both measures were able to discriminate movement related behavior or energy expenditure patterns within the broader physical activity construct.

When looking at the strengths of correlations between BPAQ indices and IPAQ-SF variables, an important finding of this study is the consistent low correlations of the leisure and sport indices of BPAQ with all IPAQ-SF outcomes. The low correlations of these two subscales with even the IPAQ moderate and vigorous activities may suggest the BPAQ did not capture well the leisure and sport activities of the participants in the present study. However, this relative lack of correspondence of BPAQ’s leisure and sport subscales with IPAQ outcomes might be a reflection of reduced variation in estimates from both subscales due to the low prevalence of sport-related leisure activities reported by the participants. Previous studies have reported similar low prevalence of sport participation and active leisure activities among urban and rural South African adults [55, 56].

The large mean difference found for total physical activity scores between methods in the present study is similar to the agreement result between overall physical activity scores from the Active Australian Survey and National Health Survey physical activity questionnaires among Australian adults [44]. Though the large mean difference indicates discrepancies due to systematic bias or measurement error between the IPAQ-SF and BPAQ estimates, the observed 95 % limits of agreement is small enough to suggest that the magnitude of bias between methods was reasonable. The high level of disagreement in our study could reflect the different scoring approach for each questionnaire [53]. However, patterns of physical activity estimates from the IPAQ-SF and BPAQ were similar and in the expected direction between subgroups of our sample. For example, at both urban and rural areas, and consistent with evidence [4–7, 13, 57, 58], men were more physically active than women on all the physical activity estimates from both methods, suggesting the inherent ability of both IPAQ-SF and BPAQ to rate similar physical activity constructs in a consistent fashion.

The sensitivity of the BPAQ to correctly classify individuals as sufficiently active according to the IPAQ category was very good but its specificity was weak. The application of this finding is that majority of individuals classified into the moderate-to high physical activity category by the BPAQ also reached the current public health recommendations threshold of sufficient physical activity as classified by IPAQ, but only a few could be correctly classified as insufficiently active (less than 150 min/week) or inactive (less than 30 min/week). This is plausible given that the IPAQ has been found to overestimate meeting the international health-related physical activity guidelines [38, 43, 59, 60], in part because it assesses all domains of activity and the guidelines are based mainly on leisure-time physical activity [33] that was not adequately captured by the BPAQ, as reflected by the poor performance of its leisure and sport indices in the present study. A possible approach to improve the specificity of BPAQ among older black South African adults is to tailor the activities on its leisure and sport subscales to be more relevant to the local population. For instance, the examples of given sporting activities like billiards, sailing, golf, rowing, rugby are not popular among older black South African adults and could be replaced with relevant and culturally appropriate sports or games of equivalent metabolic energy turnover. Presently the BPAQ captured both active and passive sedentary activities together on the leisure time subscale. Thus, to improve specificity, the leisure index of BPAQ could be refined to differentiate between leisure-time physical activities and sedentary leisure activities. Further, responses to the BPAQ leisure and sport questions could be improved by development of show cards to simplify the different examples of physical activity assessed by these subscales when administering the questionnaire.

Generally, the findings of notable disparities between physical activity estimates from the two methods clearly have implications for the physical activity epidemiology field in Africa and other LMICs where self-report method is likely to continue being the dominant mode of physical activity assessment [12, 15]. Though both questionnaires seem to rate physical activity constructs well in the present study, they differ in the specific dimensions of physical activity they assessed, the time frame of recall, and the expression of results. Thus, based on our experience we will generally recommend that the IPAQ-SF should be used for research in Africa when the goal is to conduct surveillance, monitor trends, determine prevalence and to compare within and between populations estimate of physical activity. The BPAQ should be used in studies where longer recall periods are needed due to seasonality or among low educated people in Africa for which estimation of time spend in physical activity may be poor [19].

This study had some strengths. It was conducted in a relatively large sample of adults recruited from urban and rural communities and the response rate was high, enhancing the generalizability of findings to diverse populations in Africa. It was one of the first to examine the magnitude of agreements and sensitivity of self-report physical activity measures in the African population. It is valuable to evaluate standardized questionnaires developed in high-income countries for applicability to Africa because this can enhance local relevance of evidence and interventions. Further, the participants were not aware the data would be used for a measurement study, therefore reducing respondent bias and enhancing generalizability of the findings.

The study has also some important limitations. Assessing relative validity between two self-report measures may be misleading, as correlations between methods do not mean either questionnaire is valid as they may be subject to correlated errors [9]. Additionally, studies comparing the performance of different physical activity questionnaires should acknowledge that in the lack of accelerometry data, no questionnaires are to be assumed as the ‘reference method’ [12]. It is important that future studies should simultaneously examine the agreement of both IPAQ-SF and BPAQ variables with conceptually matched accelerometetry variables. Also, the agreement between IPAQ and BPAQ would have been underestimated in our analysis because different units of measurements have been used by both methods and the repeatability of the two questionnaires was not estimated. The extent of repeatabilities of two methods of measurement limits the amount of agreement which is possible [53]. However, we believe that our findings highlighted the difficulty of inter-survey comparison and provided preliminary evidence that the use of different self-report methods for all studies may not be adequate for physical activity surveillance and monitoring in Africa.

## Conclusions

Overall, there is modest correlation but notable disparities between physical activity estimates from the IPAQ-SF and BPAQ among urban and rural Black South African adults. Our findings have implications for the choice of self-report methods in physical activity surveillance and epidemiology studies in Africa. For consistency in epidemiological data over time in Africa, utilization of IPAQ-SF and BPAQ for physical activity research should depend on the research questions and population to be studied.

## Abbreviations

BMI: Body mass index
BPAQ: Baecke physical activity questionnaire
CI: Confidence interval
IPAQ-SF: International physical activity questionnaire-short form
LMICs: Low and middle income countries
MET: Metabolic equivalent
MVPA: Moderate-to-vigorous physical activity
PURE: Prospective Urban and Rural Epidemiological study

## References

[1] World Health Organization (2015). Global status report on noncommunicable diseases 2014.

[2] Pratt M, Sarmiento OL, Montes F, Ogilvie D, Marcus BH, Perez LG (2012). The implications of megatrends in information and communication technology and transportation for changes in global physical activity. Lancet.

[3] Kohl HW, Craig CL, Lambert EV, Inoue S, Alkandari JR, Leetongin G (2012). The pandemic of physical inactivity: global action for public health. Lancet.

[4] Hallal PC, Andersen LB, Bull FC, Guthold R, Haskell W, Ekelund U (2012). Global physical activity levels: surveillance progress, pitfalls, and prospects. Lancet.

[5] Troiano RP, Berrigan D, Dodd KW, Masse LC, Tilert T, McDowell M (2008). Physical activity in the United States measured by accelerometer. Med Sci Sports Exerc.

[6] Golubic R, Martin KR, Ekelund U, Hardy R, Kuh D, Wareham N (2014). Levels of physical activity among a nationally representative sample of people in early old age: results of objective and self-reported assessments. Int J Behav Nutr Phys Act.

[7] Evenson KR, Buchner DM, Morland KB (2012). Objective measurement of physical activity and sedentary behavior among US adults aged 60 years or older. Prev Chronic Dis.

[8] Pedisic Z, Bauman A (2015). Accelerometer-based measures in physical activity surveillance: current practices and issues. Br J Sports Med.

[9] Warren JM, Ekelund U, Besson H, Mezzani A, Geladas N, Vanhees L (2010). Assessment of physical activity – a review of methodologies with reference to epidemiological research: a report of the exercise physiology section of the European Association of Cardiovascular prevention and Rehabilitation. Eur J Cardiovasc Prev Rehabil.

[10] Prince SA, Adamo KB, Hamel ME, Hardt J, Gorber SC, Tremblay M (2008). A comparison of direct versus self-report measures for assessing physical activity in adults: a systematic review. Int J Behav Nutr Phys Act.

[11] Sallis JF, Saelens BE (2000). Assessment of physical activity by self-report: Status, limitations, and future directions. Res Q Exerc Sport.

[12] Hallal PC, Matsudo S, Farias JC (2012). Measurement of physical activity by self-report in low-and middle income countries: more of the same is not enough. J Phys Act Health.

[13] Dumith MC, Hallal PC, Reis RS, Kohl HW (2011). Worldwide prevalence of physical inactivity and its association with human development index in 76 countries. Prev Med.

[14] Guthold R, Louazani SA, Riley LM, Cowan MJ, Bovet P, Damasceno A (2011). Physical activity in 22 African countries: results from WHO STEPwise approach to chronic disease risk factor surveillance. Am J Prev Med.

[15] Sobngwi E, Mbanya JC, Unwin NC, Aspray TJ, Alberti KG (2001). Development and validation of a questionnaire for the assessment of physical activity in epidemiological studies in Sub-Saharan Africa. Int J Epidemiol.

[16] Craig CL, Marshall AL, Sjostrom M, Bauman AE, Booth ML, Ainsworth BE (2003). International physical activity questionnaire: 12-country reliability and validity. Med Sci Sports Exerc.

[17] Oyeyemi AL, Umar M, Oguche F, Aliyu SU, Oyeyemi AY (2014). Accelerometer-determined physical activity and its comparison with the international physical activity questionnaire in a sample of Nigerian adults. PLoS One.

[18] Bull FC, Maslin TS, Armstrong T (2009). Global physical activity questionnaire (GPAQ): Nine country reliability and validity study. J Phys Act Health.

[19] Kruger HS, Venter CS, Steyn HS (2000). A standardized physical activity questionnaire for a population in transition: the THUSA study. Afr J Phys Health Educ Recreat Dance.

[20] Joubert J, Norman R, Lambert EV, Groeneward P, Scheider M, Bull F (2007). Estimating the burden of diseases attributable to physical inactivity in South-Africa in 2000. S Afr Med J.

[21] Kruger HS, Venter CS, Vorster HH, Margetts BM (2002). Physical inactivity is the major determinants of obesity in black women in the North West Province, South-Africa: the THUSA study. Nutrition.

[22] Kolbe-Alexander TL, Lambert EV, Harkins JB, Ekelund U (2006). Comparison of two methods of measuring physical activity in South African older adults. J Aging Phys Act.

[23] Baecke JA, Burema J, Frijters JE (1982). A short questionnaire for the measurement of habitual physical activity in epidemiological studies. Am J Clin Nutr.

[24] Ho SC, Yu R, Chan S (2015). Comparison of the modified Chinese Baecke questionnaire with a 3-day activity diary in a Hong Kong Chinese population. Asia Pac J Public Health.

[25] Hertog EM, Monninkhof EM, Schouten EG, Peeters PHM, Scuit AJ (2008). Validity of the modified Baecke questionnaire: comparison with energy expenditure according to the doubly labeled water method. Int J Behav Nutr Phys Act.

[26] Ono R, Hirata S, Yamada M, Nishiyama T, Kurosaka M, Tamura Y (2007). Reliability and validity of the Baecke physical activity questionnaire in adult women with hip disorders. BMC Musculoskelet Dis.

[27] Philippaerts MR, Lefevre J (1998). Reliability and validity of three physical activity questionnaires in Flemish males. Am J Epidemiol.

[28] Yu R, Yau F, Ho SC, Woo J (2013). Longitudinal changes in physical activity levels over 5 years and relationship to cardiorespiratory fitness in Chinese midlife women. J Sports Med Phys Fitness.

[29] Autenrieth CS, Evenson KR, Yatsuya H, Shahar E, Baggett C, Rosamond WD (2013). Association between physical activity and risk of stroke subtypes: the athreosclerosis risk in communities (ARIC) study. Neuroepidemiology.

[30] Teo K, Lear S, Islam S, Mony P, Degham M, Li W (2013). Prevalence of a healthy lifestyle among individuals with cardiovascular diseases in high-, middle- and low-income countries. The Prospective Urban Rural Epidemiology (PURE) Study. JAMA.

[31] Tehard B, Saris WHM, Astrup A, Martinez JA, Taylor MA, Barbe P (2005). Comparison of two physical activity questionnaires in obese subjects: The NUGENOB Study. Med Sci Sports Exerc.

[32] World Health Organization (2010). Global recommendations on physical activity for health.

[33] Garber CE, Blissmer B, Deschenes MR, Franklin BA, Lamonte MJ, Lee IM (2011). American College of Sports Medicine position stand. Quality and quantity of exercise for developing and maintaining cardiorespiratory, musculoskeletal and neuromuscular fitness in apparently healthy adults: Guidance for prescribing exercise. Med Sci Sports Exerc.

[34] Ekelund U, Sepp H, Brage S, Becker W, Jakes R, Hennings M (2006). Criterion-related validity of the last 7-day, short form of the international physical activity questionnaire in Swedish adults. Public Health Nutr.

[35] Chun MY (2012). Validity and reliability of Korean version of international physical activity questionnaire short form in the elderly. Korean J Fam Med.

[36] Kurtze N, Rangul V, Hustveldt B (2008). Reliability and validity of the international physical activity questionnaire in the Nord-Trondelag Health Study (HUNT) population of men. BMC Med Res Methodol.

[37] Oyeyemi AL, Oyeyemi AY, Adegoke BO, Oyetoke FO, Aliyu HN, Aliyu SU (2011). Cross cultural adaptation of the International Physical Activity Questionnaire: reliability and validity of the Hausa version in Nigeria. BMC Med Res Methodol.

[38] Bauman A, Ainsworth BE, Bull F, Craig CL, Hagstromer M, Sallis JF (2009). Progress and pitfalls in the use of the international physical activity questionnaire (IPAQ) for adult physical activity surveillance. J Phys Act Health.

[39] Bauman A, Bull FC, Craig CL, Chey T, Ainsworth BE, Sallis JF (2009). The International prevalence study on physical activity: results from 20 countries. Int J Behav Nutr Phys Act.

[40] Sallis JF, Bowles HR, Bauman A, Ainsworth BE, Bull FC, Craig CL (2009). Neighborhood environment and physical activity among adults in 11 countries. Am J Prev Med.

[41] Kerr J, Emond JA, Badland H, Reis R, Sarmiento O, Carlson J (2016). Perceived neighborhood environmental attributes associated with walking and cycling for transport among adult residents of 17 cities in 12 countries: the IPEN study. Environ Health Perspect.

[42] Hallal PC, Gomez LF, Parra DC, Lobelo F, Mosquera J, Florindo AA (2010). Lessons learned after 10 years of IPAQ use in Brazil and Colombia. J Phys Act Health.

[43] Ainsworth BE, Macera CA, Jones DA, Reis JP, Addy CL, Bowles HR (2006). Comparison of the 2001 BRFSS and the IPAQ physical activity questionnaires. Med Sci Sports Exerc.

[44] Brown W, Bauman A, Chey T, Trost S, Mummery K (2004). Comparison of surveys used to measure physical activity. Aust N Z J Public Health.

[45] Teo K, Chow CK, Vaz M, Rangarajan S, Yusuf S (2009). The Prospective Urban Rural Epidemiology (PURE) study: examining the impact of societal influences on chronic non communicable diseases in low-, middle-, and high-income countries. Am Heart J.

[46] Yusuf S, Islam S, Chow CK, Rangarajan S, Dagenais G, Diaz R (2011). Use of secondary prevention drugs for cardiovascular disease in the community in high-income, middle-income, and low-income countries (the PURE Study): a prospective epidemiological survey. Lancet.

[47] International Physical Activity Questionnaire (IPAQ) (2005). Guidelines for data processing and analysis of the international physical activity questionnaire.

[48] Sallis JF, Bull FC, Guthold R, Heath GW, Inoue S, Kelly P, et al. Progress in physical activity over the Olympic quadrennium. Lancet. 2016. http://dx.doi.org/10.1016/S0140-6736(16)30581-5. [Epub ahead of print]. 10.1016/S0140-6736(16)30581-5 27475270

[49] McGraw KO, Wong SP (1996). Forming inferences about some intraclass correlation coefficients. Psychol Methods.

[50] Muller R, Buttner P (1994). A critical discussion of intraclass correlation coefficients. Stat Med.

[51] Friendenreich CM, Courneya KS, Neilson HK, Matthews CE, Willis G, Irwin M (2006). Reliability and validity of the past year total physical activity questionnaire. Am J Epidemiol.

[52] Cohen J (1988). Statistical power analysis for the behavioral sciences.

[53] Bland JM, Altman DG (1999). Measuring agreements in methods comparison studies. Stat Methods Med Res.

[54] Landis JR, Koch GG (1977). The measurement of observer agreement for categorical data. Biometrics.

[55] Kruger HS, Venter CS, Vorster HH (2002). Physical inactivity as a risk factor for cardiovascular disease in communities undergoing rural to urban transition: the THUSA study. Cardiovasc J South Afr.

[56] Sparling PB, Noakes TD, Steyn K, Jordaan E, Jooste PL, Bourne LT, Badenhorst C (1994). Level of physical activity and CHD risk factors in black South African men. Med Sci Sports Exerc.

[57] Bauman AE, Reis RS, Sallis JF, Wells JC, Loos RJF, Martin BW (2012). Correlates of physical activity: Why are some people physically active and others not?. Lancet.

[58] Guthold R, Ono T, Strong KL, Chatterji S, Morabia A (2008). Worldwide variability in physical inactivity, a 51-country survey. Am J Prev Med.

[59] Rzewnicki R, Vanden Auweele Y, De Bourdeaudhuij I (2003). Addressing overreporting on the International Physical Activity Questionnaire (IPAQ) telephone survey with a population sample. Public Health Nutr.

[60] Johnson-Kozlow M, Sallis JF, Gilpin EA, Rock CL, Pierce JP (2006). Comparative validation of the IPAQ and the 7-Day PAR among women diagnosed with breast cancer. Int J Behav Nutr Phys Act.

